# Association Between Discrimination and Depressive Symptoms Among Hispanic or Latino Adults During the COVID-19 Pandemic: Cross-Sectional Study

**DOI:** 10.2196/48076

**Published:** 2024-06-06

**Authors:** Cameron K Ormiston, Kevin Villalobos, Francisco Alejandro Montiel Ishino, Faustine Williams

**Affiliations:** 1 National Institute on Minority Health and Health Disparities National Institutes of Health Rockville, MD United States; 2 Department of Medical Education Icahn School of Medicine at Mount Sinai New York, NY United States; 3 National Institute of Environmental Health Sciences National Institutes of Health Durham, NC United States

**Keywords:** depressive symptoms, everyday discrimination, COVID-19 pandemic, Hispanic and Latino, immigrant health

## Abstract

**Background:**

Discrimination and xenophobia toward Hispanic and Latino communities increased during the COVID-19 pandemic, likely inflicting significant harm on the mental health of Hispanic and Latino individuals. Pandemic-related financial and social instability has disproportionately affected Hispanic and Latino communities, potentially compounding existing disparities and worsening mental health.

**Objective:**

This study aims to examine the association between discrimination and depressive symptoms during the COVID-19 pandemic among a national sample of Hispanic and Latino adults.

**Methods:**

Data from a 116-item web-based nationally distributed survey from May 2021 to January 2022 were analyzed. The sample (N=1181) was restricted to Hispanic or Latino (Mexican or Mexican American, Puerto Rican; Cuban or Cuban American, Central or South American, and Dominican or another Hispanic or Latino ethnicity) adults. Depression symptoms were assessed using the 2-item Patient Health Questionnaire. Discrimination was assessed using the 5-item Everyday Discrimination Scale. A multinomial logistic regression with a block entry model was used to assess the relationship between discrimination and the likelihood of depressive symptoms, as well as examine how controls and covariates affected the relationship of interest.

**Results:**

Mexican or Mexican American adults comprised the largest proportion of the sample (533/1181, 45.13%), followed by Central or South American (204/1181, 17.3%), Puerto Rican (189/1181, 16%), Dominican or another Hispanic or Latino ethnicity (172/1181, 14.6%), and Cuban or Cuban American (83/1181, 7.03%). Approximately 31.26% (367/1181) of the sample had depressive symptoms. Regarding discrimination, 54.56% (634/1181) reported experiencing some form of discrimination. Compared with those who did not experience discrimination, those who experienced discrimination had almost 230% higher odds of depressive symptoms (adjusted odds ratio [AOR] 3.31, 95% CI 2.42-4.54). Also, we observed that sociodemographic factors such as age and gender were significant. Compared with participants aged 56 years and older, participants aged 18-35 years and those aged 36-55 years had increased odds of having depressive symptoms (AOR 3.83, 95% CI 2.13-6.90 and AOR 3.10, 95% CI 1.74-5.51, respectively). Women had higher odds of having depressive symptoms (AOR 1.67, 95% CI 1.23-2.30) than men. Respondents with an annual income of less than US $25,000 (AOR 2.14, 95% CI 1.34-3.41) and US $25,000 to less than US $35,000 (AOR 1.89, 95% CI 1.17-3.06) had higher odds of depressive symptoms than those with an annual income of US $50,000 to less than US $75,000.

**Conclusions:**

Our findings provide significant importance especially when considering the compounding, numerous socioeconomic challenges stemming from the pandemic that disproportionately impact the Hispanic and Latino communities. These challenges include rising xenophobia and tensions against immigrants, inadequate access to mental health resources for Hispanic and Latino individuals, and existing hesitations toward seeking mental health services among this population. Ultimately, these findings can serve as a foundation for promoting health equity.

## Introduction

Mental health conditions are a major public health concern in the United States, with 20% of US adults (ie, 52.9 million people) experiencing mental illness annually [1]. Risk factors for mental health conditions include having chronic diseases, experiencing homelessness, financial and food insecurity, substance use, racism and discrimination, and stressful life events [2-5]. The prevalence of mental health conditions also varies by sociodemographic factors such as gender, race and ethnicity, and income [2,6-9]. Although data suggest that racial and ethnic minorities have lower rates of mental health conditions than White individuals, these trends may be a result of higher rates of misdiagnosis or underdiagnosis of mental health conditions among non–White populations [6,10,11]. Furthermore, the impact of mental health symptoms is more far-reaching and drastic among racial and ethnic minoritized communities [6,10]. Despite having a lower lifetime risk for mood and anxiety disorders, Hispanic and non-Hispanic Black adults are more likely to have persistent disorders than non-Hispanic White adults [12].

In addition, barriers to mental health care access are more pronounced among racial and ethnic minoritized communities. For example, disability burden due to inadequate mental health services and unmet needs are disproportionately higher among racial and ethnic minorities [13]. Among adults with moderate or severe anxiety or depression in 2019, 53% of Black adults and 40% of Hispanic adults reported that they were not receiving treatment [14]. Lower use and poorer access to psychological services among underrepresented and underserved racial and ethnic groups (eg, Black and African American communities; Hispanic and Latino communities) stem from socioeconomic inequities that are compounded by stigma, financial barriers, structural racism, and lack of representation in health care [5,10,15,16]. Moreover, historical and current discrimination, as well as abuse by the health care system, discordance between providers and patients, and lack of cultural humility and empathy among providers perpetuates apprehension to seek care and worsens mental health care access and use [5,10,15,16].

The COVID-19 pandemic has had especially drastic mental health consequences, with the prevalence of depression and anxiety being nearly 4-fold higher during the pandemic (2020) than before (2019) [17]. Concurrent with the increase in mental health burden in the United States was an increase in anti-immigrant sentiment and misinformation, racism, discrimination, exploitation, and xenophobia targeted toward Hispanic and Latino groups [18]. Exclusion of immigrants, 44% of whom are Hispanic or Latino (ie, two-thirds of Hispanic and Latino individuals in the United States are born outside the United States), from COVID-19 relief bills has likely further entrenched inequities for immigrant, Hispanic, and Latino communities in the United States [19,20]. Moreover, Hispanic and Latino communities have been disproportionately affected by COVID-19, having higher rates of COVID-19 infection, hospitalization, and mortality [19,21]. Data on layoffs due to the pandemic also show that Hispanic and Latino adults had the highest probability of being laid off compared with their Black, Asian, and White counterparts [22]. Thus, the increase in discrimination toward Hispanic and Latino groups combined with preexisting low psychological service use rates and worsening financial and social disparities is likely to have major, synergistic effects on the acute and long-term mental health of the Hispanic and Latino community. In fact, data as recent as February 2023 from the Household Pulse Survey show that Hispanic or Latino adults have the highest prevalence of depressive symptoms among all racial and ethnic groups [23]. Hispanic and Latino adults also reported a higher prevalence of depressive symptoms and a higher unmet need for mental health services compared with non-Hispanic Asian, non-Hispanic Black, and non-Hispanic White adults during the pandemic (March 2020, December 2020, and January 2021) [4,17,24,25]. Moreover, a prepandemic meta-analysis of 333 studies reported that discrimination may incur greater mental health tolls for Latino adults compared to other racial and ethnic groups, indicating that recent increases in discrimination may have particularly drastic effects on Hispanic and Latino psychological outcomes [26].

Importantly, the heterogeneity of the US Hispanic or Latino population must be considered, with prepandemic research showing differences in psychological distress, depression, and anxiety across acculturation and Hispanic or Latino ethnicity [27-32]. However, it remains to be seen whether these differences are present during the pandemic. To address these knowledge gaps, we examined the association of discrimination and depressive symptoms during the COVID-19 pandemic among a national sample of Hispanic and Latino adults that was further analyzed by Hispanic or Latino ethnicity group. We hypothesize that (1) discrimination will be associated with higher odds of depressive symptoms and (2) differences will be observed in the odds of depressive symptoms across Hispanic or Latino ethnicity group.

## Methods

### Study Participants

Data from “Understanding the Impact of the Novel Coronavirus (COVID-19) and Social Distancing on Physical and Psychosocial (Mental) Health and Chronic Diseases” were analyzed. This was a 116-item web-based survey that was nationally distributed in the United States from May 2021 to January 2022. Qualtrics, LLC conducted recruitment and survey distribution using a national survey panel. The survey was in English only. For more details, see the study by Montiel Ishino et al [33]. Analysis was restricted to Hispanic or Latino (ie, Mexican or Mexican American; Puerto Rican, Cuban or Cuban American, Central or South American, and Dominican or another Hispanic or Latino ethnicity) adults, which included both foreign-born and US-born groups. The final sample size was 1181.

### Outcome Variable: Depressive Symptoms

Depressive symptoms were assessed using the Patient Health Questionnaire-2 [34]. Two items were used to assess whether “Over the last 2-week period how often were you bothered by the following problems?”: (1) “Little interest or pleasure in doing things”; and (2) Feeling down, depressed or hopeless.” Responses to these items included (1) “not at all” for a score of 0; (2) “several days” for a score of 1; (3) “more than half the days” for a score of 2; and (4) “nearly every day” for a score of 3. Scores were summed for a range of 0-6, where a score of 0-2 (reference category) indicates no depressive symptoms and a score of 3 or more indicates present depressive symptoms [34]. The Patient Health Questionnaire-2 has been validated by both Spanish- and English-speaking Hispanic and Latino adults [34-37].

### Predictor Variable: Everyday Discrimination

The short version of the Everyday Discrimination Scale was used [38]. Items of the short version assessed whether “In your day-to-day life how often have any of the following things happened to you?”: (1) You are treated with less courtesy or respect than other people. (2) You receive poorer service than other people at restaurants or stores. (3) People act as if they think you are not smart. (4) People act as if they are afraid of you. (5) You are threatened or harassed. Responses to the 5 items were (1) never; (2) monthly to weekly; and (3) multiple times a week to daily. A composite score was created to compare participants who had never experienced any discrimination with those with experiences of discrimination.

### Acculturation and Sociodemographic Covariates and Controls

All items were self-reported. Acculturation factors included years in the United States (ie, less than 10 years and 10 or more years) and English-speaking proficiency level (ie, none or poor, fairly well, well, or very well). Self-reported sociodemographic factors included the following: age group (ie, 18-35 years, 36-55 years, and 56 years of age and older); gender (ie, man, woman, and another gender category that included responses for nonbinary, transgender, or responses provided through fill in the blank); marital status (ie, divorced or separated, married or living with a partner, never been married, or widowed); educational attainment (ie, categorized by self-reported highest schooling that included less than high school, did not attend school, elementary level education, more than elementary to junior high school, or some high school; high school diploma or GED; some college or vocational or technical schooling; bachelor’s degree; and master’s degree or above that includes doctoral, professional, or postgraduate degree); and annual household income (ie, US <$25,000, US $25,000 to US $34,999, US $35,000 to US $49,999, US $50,000 to US $74,999, and US ≥$75,000).

### Statistical Analysis

We used a multinomial logistic regression with a block entry model to assess the relationship between discrimination and the likelihood of depressive symptoms, as well as to examine how controls and covariates affected the relationship of interest. All findings are reported in odds ratios (ORs) or adjusted odds ratios (AORs) using 95% CI and *P* value at ≤.05 statistical level of significance. All analyses were conducted using Stata/MP (version 16.1; StataCorp).

### Ethical Considerations

The NIH Intramural Research Program institutional review board, Human Research Protection Program, and Office of Human Subjects Research Protections reviewed this study’s research protocol, determined that our protocol did not involve human participants and granted an exemption on December 23, 2020 (IRB#000308). For full information on ethical considerations for this study, see the study by Ishino et al [33]. Qualtrics recruited participants, and web-based informed consent was obtained from each participant prior to filling out the survey. Participants were assured that responses were confidential and were reminded that the study was voluntary, and they could opt out of the survey or skip any questions without repercussions. All answers were kept confidential, and no personal identifiers were shared with the National Institute on Minority Health and Health Disparities research team, which participants were also made aware of. A US $10 gift card was given to each participant after the completion of their survey, which took approximately 30 minutes to complete.

## Results

### Sample Characteristics

The largest subpopulation among the sample was Mexican or Mexican American (533/1181, 45.13%), followed by Central or South American (204/1181, 17.3%), Puerto Rican (189/1181, 16%), Dominican or another Hispanic or Latino ethnicity (172/1181, 14.6%), and Cuban or Cuban American (83/1181, 7.03%; Table 1). Depressive symptoms were reported in 31.26% (367/1181) of the sample and 54.56% (634/1181) reported experiencing some form of discrimination. Most of the participants reported living in the United States for 10 years or more (900/1181, 76.53%), spoke English very well (832/1181, 70.63%), were 18-35 years of age (541/1181, 49.54%), and were women (728/1181, 61.75%). About a quarter of participants (290/1181, 24.83%) reported having a household income of less than US $25,000 (Table 1).

**Table 1:** Descriptive characteristics of the sample (N=1181)^a^.

		Values, n (%)
**Depressive symptoms**		
	Present depressive symptoms	367 (31.26)
**Discrimination**		
	Experienced discrimination	634 (54.56)
**Years in the United States**		
	≥10	900 (76.53)
**English-speaking proficiency**		
	None or poor	60 (5.09)
	Fairly well	94 (7.98)
	Well	192 (16.30)
	Very well	832 (70.63)
**Age (years)**		
	18-35	541 (49.54)
	36-55	366 (33.52)
	56 and older	185 (16.94)
**Gender**		
	Another gender	32 (2.71)
	Man	419 (35.53)
	Woman	728 (61.75)
**Hispanic or Latino ethnicity**		
	Central or South American	204 (17.27)
	Cuban or Cuban American	83 (7.03)
	Dominican or another Hispanic or Latino ethnicity	172 (14.56)
	Mexican or Mexican American	533 (45.13)
	Puerto Rican	189 (16)
**Marital status**		
	Divorced or separated	106 (9.01)
	Married or living with a partner	617 (52.47)
	Never been married	425 (36.14)
	Widowed	28 (2.38)
**Education**		
	Less than high school	36 (3.06)
	High school or GED^b^	368 (31.24)
	Some college, vocational, or technical	364 (30.9)
	Bachelor’s degree	280 (23.77)
	Master’s degree or above	130 (11.04)
**Household income (US $)**		
	<$25,000	290 (24.83)
	$25,000 to <$35,000	208 (17.81)
	$35,000 to <$50,000	190 (16.27)
	$50,000 to <$75,000	235 (20.12)
	≥$75,000	245 (20.98)

^a^Some category values do not add to the total (N) due to missing numbers.

^b^GED: General Education Development.

### Multinomial Logistic Regression

Model 1 (the unadjusted model) found that there was a significant relationship between discrimination and the likelihood of depressive symptoms. In model 2, we then included acculturation factors, where years in the United States were found to be significant. In model 3, the comprehensive model that was selected for interpretation included the variables from the aforementioned models in addition to sociodemographic variables. Compared with those who did not experience discrimination, those who experienced discrimination had almost 230% higher odds of depressive symptoms (AOR 3.31, 95% CI 2.42-4.54). In addition, we observed that sociodemographic factors such as age and gender were significant. Participants aged 18-35 years of age and 36-55 years of age had increased odds of having depressive symptoms (AOR 3.83, 95% CI 2.13-6.90 and AOR 3.10, 95% CI 1.74-5.51, respectively) when compared with participants 56 years of age and older. Regarding gender, women compared with men had higher odds of having depressive symptoms (AOR 1.67, 95% CI 1.23-2.30). Respondents with an annual income of less than US $25,000 (AOR 2.14, 95% CI 1.34-3.41) and US $25,000 to less than US $35,000 (AOR 1.89, 95% CI 1.17-3.06) had higher odds of depressive symptoms than those with an annual income of US $50,000 to less than US $75,000 (Table 2).

**Table 2:** OR^a^ and CIs for associations between depressive symptoms and covariates^b^.

		Model 1, OR (95% CI)	Model 2, AOR^c^ (95% CI)	Model 3, AOR^c^ (95% CI)
**Discrimination**				
	Experienced discrimination	*3.66 (2.78-4.82)^d^*	*3.55 (2.69-4.70)*	*3.31 (2.42-4.54)*
	Experienced no discrimination	Reference	Reference	Reference
**Years in the United States**				
	<10	N/A^e^	*1.46 (1.04-2.05)*	1.34 (0.90-1.98)
	≥10	N/A	Reference	Reference
**English-speaking proficiency**				
	None or poor	N/A	0.78 (0.41-1.46)	0.71 (0.34-1.49)
	Fairly well	N/A	*0.58 (0.34-0.99)*	0.60 (0.34-1.08)
	Well	N/A	0.89 (0.61-1.30)	1.04 (0.68-1.58)
	Very well	N/A	Reference	Reference
**Age (years)**				
	18-35	N/A	N/A	*3.83 (2.13-6.90)*
	36-55	N/A	N/A	*3.10 (1.74-5.51)*
	56 and older	N/A	N/A	Reference
**Gender**				
	Another gender identity	N/A	N/A	1.50 (0.65-3.46)
	Man	N/A	N/A	Reference
	Woman	N/A	N/A	*1.67 (1.23-2.30)*
**Hispanic or Latino ethnicity**				
	Central or South American	N/A	N/A	0.73 (0.43-1.22)
	Cuban or Cuban American	N/A	N/A	1.44 (0.71-2.91)
	Dominican or another Hispanic or Latino ethnicity	N/A	N/A	Reference
	Mexican or Mexican American	N/A	N/A	1.01 (0.66-1.55)
	Puerto Rican	N/A	N/A	1.22 (0.72-2.05)
**Marital status**				
	Divorced or separated	N/A	N/A	1.05 (0.60-1.84)
	Married or living with a partner	N/A	N/A	0.99 (0.71-1.38)
	Never been married	N/A	N/A	Reference
	Widowed	N/A	N/A	1.72 (0.58-5.09)
**Education**				
	Less than high school	N/A	N/A	0.77 (0.29-2.00)
	High school or general educational diploma	N/A	N/A	1.22 (0.70-2.14)
	Some college, vocational, or technical	N/A	N/A	1.06 (0.62-1.83)
	Bachelor’s degree	N/A	N/A	0.71 (0.41-1.23)
	Master’s degree or above	N/A	N/A	Reference
**Household income (US $)**				
	<$25,000	N/A	N/A	*2.14 (1.34-3.41)*
	$25,000 to <$35,000	N/A	N/A	*1.89 (1.17-3.06)*
	$35,000 to <$50,000	N/A	N/A	1.35 (0.83-2.20)
	$50,000 to <$75,000	N/A	N/A	Reference
	≥$75,000	N/A	N/A	1.44 (0.89-2.33)

^a^OR: odds ratio.

^b^Covariates included years in the United States, English-speaking proficiency level, age, gender, marital status, educational attainment, and annual household income.

^c^AOR: adjusted odds ratio.

^d^Values in italics indicate statistical significance level at *P*≤.05.

^e^N/A: not applicable.

## Discussion

### Principal Findings

We found a significant association between discrimination and depressive symptoms that persisted after adjusting for acculturation and sociodemographic factors, indicating that discrimination cannot be overlooked among Hispanic and Latino communities during the pandemic. This relationship is particularly important given discrimination’s role in perpetuating systemic inequities and socioeconomic disparities [39]. For instance, research predating the pandemic reported that discrimination toward Latino communities significantly limits job opportunities, income, housing, and education [40,41]. Discrimination has also been shown to increase substance use disorders, chronic disease, and blood pressure [42]. All these discrimination-related adverse life and health outcomes were risk factors for poor physical and mental health during the pandemic [43]. Thus, when placing our findings in the context of a pandemic that has caused greater financial and social instability for racial and ethnic minoritized communities, the need to address the deleterious effects of discrimination on the mental health of Hispanic and Latino communities is urgently salient. Ultimately, our findings mirror research showing that discrimination is associated with depressive symptoms and other adverse psychological outcomes both predating [26,40,42,44] and during the COVID-19 pandemic [45-47].

Contrary to our hypothesis, we did not find differences in the odds of depressive symptoms by Hispanic or Latino subgroup. This may be because we adjusted for sociodemographic and migration factors, which have been shown to attenuate differences in anxiety and psychiatric disorders by Hispanic or Latino subgroup [48,49]. Furthermore, our findings may be an indication that the pandemic was detrimental to mental health irrespective of subgroup given the prevalence of discrimination and depressive symptoms was exceedingly high and similar across all subgroups. Thus, future research should examine whether discrimination impacts depression symptoms differently by Hispanic or Latino subgroup during the pandemic and beyond. For instance, Held and Lee’s [49] study about discrimination and mental health among Latino adults reported that discrimination was more likely to have an effect on the odds of having a psychiatric disorder among Mexican adults than among Puerto Rican adults, and there was no difference between Cubans and Puerto Ricans. However, no further investigation or comparison regarding other Hispanic or Latino subgroups was performed.

This study also found that younger respondents (18-35 and 36-55 years of age) were more likely to report depression symptoms than older respondents (56 years of age and older). This finding is discordant with prepandemic literature, which has typically reported a higher risk of depression and levels of distress among older Latino adults [50]. Explanations for these trends lie within the longer time spent in the United States being linked with loss of cultural values that may be protective against depression and distress, and longer exposure to discrimination and acculturative stress [50,51]. Our findings align with previous research conducted during the pandemic, which has shown a consistent pattern in the United States of an inverse relationship between age and mental health symptoms, including psychological distress and depressive disorder [52,53]. Furthermore, a web-based study using a national sample of 124 Hispanic and Latino adults found that younger age was associated with poorer overall mental health during the COVID-19 pandemic [54]. These findings may be explained by younger Latino individuals (younger than 50 years) being more likely to report experiencing discrimination during the pandemic, according to a March 2021 report from the Pew Research Center, which may translate to greater odds of mental health symptoms [55].

In our study, women had higher odds of depressive symptoms than men, which is consistent with prior studies [53,56-59]. Multiple explanations for this association exist, including Latina women having greater internalizing behaviors (ie, anxiety and mood disorders), psychological distress, depressive responses to discrimination and family conflict, and experiencing greater levels of acculturative stress than Latino men [56-58,60]. Furthermore, traditional Latino cultural values such as *machismo* and *marianismo* may increase the odds of depressive symptoms among Latina women compared with men [59]. In addition, discrimination is linked to greater family conflict among Latino adults, although whether gender modifies this association is unclear and requires further study [61]. These pathways for distress and depression may be exacerbated during the pandemic, especially given that Hispanic and Latino families have faced significantly more economic and health-related hardships than Asian and White families across the income spectrum [62]. Also, among Hispanic and Latina parents, women more than men report childcare during the pandemic as being very or somewhat difficult, suggesting that the burden of family responsibilities and childcare may be higher among Latina women [63]. Furthermore, Latina women are overrepresented in unemployment and layoffs during the pandemic, which is likely to worsen financial instability and inequities [22,64]. The disproportionate burdens of stressors and risk factors may manifest into worse depressive symptoms among Latina women as seen in our study. Finally, this study found that individuals with lower income had increased odds of depressive symptoms, which is consistent with prior research showing that lower-income groups are more affected by national financial and public health crises [65-67]. Low-income Latino individuals also face numerous structural and systemic barriers to well-being, such as segregation, food deserts, limited job opportunities, and overcrowded housing—all of which can contribute to worse mental health [68-70]. Moreover, low-income and Latino communities have a heightened risk of COVID-19 incidence and severe symptoms [21,68,71]. Thus, this constellation of factors may also contribute to their higher odds of depressive symptoms.

### Limitations

Given this study is cross-sectional, directionality and causality cannot be established. Also, Dominicans and individuals who indicate “Another Hispanic, Latino, or Spanish origin” were combined due to small sample sizes. Thus, the association between discrimination and depressive symptoms among Dominicans could not be elucidated and may be obscured. In addition, the survey was conducted in English and web-based only, meaning individuals with limited English proficiency or who do not have access to the internet may not be captured and are underrepresented. Another limitation is the survey was administered over a 6-month period when vaccines became more available and some restrictions were lifted, meaning answer choices may differ depending on when the survey was completed. It should also be noted that the 5-item Everyday Discrimination Scale has not been previously validated among Hispanic and Latino adults, which may affect our results.

### Conclusions

Using a national sample of Hispanic and Latino adults, we found that discrimination was associated with depressive symptoms during the COVID-19 pandemic. We further found elevated odds of depressive symptoms among women, individuals younger than 56 years, and lower-income adults—highlighting potentially at-risk groups. Our results hold significant relevance due to the numerous pandemic-related socioeconomic crises that disproportionately impact Latino communities, increased xenophobia and tension toward immigrants, inadequate resources to address mental health care demands among Hispanic and Latino communities, and existing hesitancy in accessing mental health services in these communities. The findings of this study serve as compelling evidence of the poor depression outcomes experienced by Hispanic and Latino communities. This study can serve as an important foundational step in promoting health equity for these at-risk groups.

## Abbreviations

AOR: adjusted odds ratio
OR: odds ratio

## Data Availability

The data are available by requesting FW per the new NIH Data Management and Sharing Agreement plan.

## References

[1] (2022). Mental health by the numbers. National Alliance on Mental Illness.

[2] Yu B, Zhang X, Wang C, Sun M, Jin L, Liu X (2020). Trends in depression among adults in the United States, NHANES 2005-2016. J Affect Disord.

[3] (2023). About mental health issues. Government of Western Australia Mental Health Commission.

[4] McKnight-Eily LR, Okoro CA, Strine TW, Verlenden J, Hollis NTD, Njai R, Mitchell EW, Board A, Puddy R, Thomas C (2021). Racial and ethnic disparities in the prevalence of stress and worry, mental health conditions, and increased substance use among adults during the covid-19 pandemic—United States, April and May 2020. MMWR Morb Mortal Wkly Rep.

[5] Mensah M, Ogbu-Nwobodo L, Shim RS (2021). Racism and mental health equity: history repeating itself. Psychiatr Serv.

[6] Bailey RK, Mokonogho J, Kumar A (2019). Racial and ethnic differences in depression: current perspectives. Neuropsychiatr Dis Treat.

[7] Sareen J, Afifi TO, McMillan KA, Asmundson GJG (2011). Relationship between household income and mental disorders: findings from a population-based longitudinal study. Arch Gen Psychiatry.

[8] Brody DJ, Pratt LA, Hughes JP (2018). Prevalence of depression among adults aged 20 and over: United States, 2013-2016. NCHS Data Brief.

[9] Hargrove TW, Halpern CT, Gaydosh L, Hussey JM, Whitsel EA, Dole N, Hummer RA, Harris KM (2020). Race/ethnicity, gender, and trajectories of depressive symptoms across early- and mid-life among the add health cohort. J Racial Ethn Health Disparities.

[10] (2017). Mental health disparities: diverse populations. American Psychiatric Association.

[11] (2020). Racial disparities in diagnosis and treatment of major depression. Blue Cross Blue Shield Association.

[12] Breslau J, Kendler KS, Su M, Gaxiola-Aguilar S, Kessler RC (2005). Lifetime risk and persistence of psychiatric disorders across ethnic groups in the United States. Psychol Med.

[13] Office of the Surgeon General (US), Center for Mental Health Services (US), National Institute of Mental Health (U.S.) (2001). Mental Health : Culture, Race, and Ethnicity: A Supplement to Mental Health: A Report of the Surgeon General.

[14] Panchal N, Saunders H, Ndugga N, Kaiser Family Foundation (2022). Five key findings on mental health and substance use disorders by race/ethnicity.

[15] Dedania R, Gonzales G (2019). Disparities in access to health care among US-born and foreign-born US adults by mental health status, 2013-2016. Am J Public Health.

[16] Kyere E, Fukui S (2023). Structural racism, workforce diversity, and mental health disparities: a critical review. J Racial Ethn Health Disparities.

[17] Ettman CK, Abdalla SM, Cohen GH, Sampson L, Vivier PM, Galea S (2020). Prevalence of depression symptoms in US adults before and during the COVID-19 pandemic. JAMA Netw Open.

[18] Garcini LM, Rosenfeld J, Kneese G, Bondurant RG, Kanzler KE (2022). Dealing with distress from the COVID-19 pandemic: mental health stressors and coping strategies in vulnerable latinx communities. Health Soc Care Community.

[19] Đoàn LN, Chong SK, Misra S, Kwon SC, Yi SS (2021). Immigrant communities and COVID-19: strengthening the public health response. Am J Public Health.

[20] Krogstad JM, Passel JS, Moslimani M, Noe-Bustamamante L (2022). Key facts about U.S. latinos for national hispanic heritage month. Pew Research Center.

[21] Hill L, Artiga S, Kaiser Family Foundation (2022). COVID-19 cases and deaths by race/ethnicity: current data and changes over time.

[22] Dias FA (2021). The racial gap in employment and layoffs during COVID-19 in the United States: a visualization. Socius.

[23] US Census Bureau, National Center for Health Statistics (2023). Anxiety and depression. National Center for Health Statistics.

[24] Vahratian A, Blumberg SJ, Terlizzi EP, Schiller JS (2021). Symptoms of anxiety or depressive disorder and use of mental health care among adults during the COVID-19 pandemic—United States, August 2020-February 2021. MMWR Morb Mortal Wkly Rep.

[25] Fitzpatrick KM, Harris C, Drawve G (2020). Living in the midst of fear: depressive symptomatology among US adults during the COVID-19 pandemic. Depress Anxiety.

[26] Paradies Y, Ben J, Denson N, Elias A, Priest N, Pieterse A, Gupta A, Kelaher M, Gee G (2015). Racism as a determinant of health: a systematic review and meta-analysis. PLoS One.

[27] Perreira KM, Gotman N, Isasi CR, Arguelles W, Castañeda SF, Daviglus ML, Giachello AL, Gonzalez P, Penedo FJ, Salgado H, Wassertheil-Smoller S (2015). Mental health and exposure to the United States: key correlates from the hispanic community health study of latinos. J Nerv Ment Dis.

[28] Alegría M, Canino G, Shrout PE, Woo M, Duan N, Vila D, Torres M, Chen CN, Meng XL (2008). Prevalence of mental illness in immigrant and non-immigrant U.S. Latino groups. Am J Psychiatry.

[29] Lee S, Held ML (2015). Variation in mental health service use among U.S. Latinos by place of origin and service provider type. Psychiatr Serv.

[30] Breslau J, Aguilar-Gaxiola S, Borges G, Castilla-Puentes RC, Kendler KS, Medina-Mora ME, Su M, Kessler RC (2007). Mental disorders among english-speaking Mexican immigrants to the US compared to a national sample of Mexicans. Psychiatry Res.

[31] Breslau J, Borges G, Hagar Y, Tancredi D, Gilman S (2009). Immigration to the USA and risk for mood and anxiety disorders: variation by origin and age at immigration. Psychol Med.

[32] Keyes KM, Martins SS, Hatzenbuehler ML, Blanco C, Bates LM, Hasin DS (2012). Mental health service utilization for psychiatric disorders among Latinos living in the United States: the role of ethnic subgroup, ethnic identity, and language/social preferences. Soc Psychiatry Psychiatr Epidemiol.

[33] Ishino FAM, Villalobos K, Williams F (2023). Substance use from social distancing and isolation by US nativity during the time of COVID-19: cross-sectional study. JMIR Public Health Surveill.

[34] Kroenke K, Spitzer RL, Williams JBW (2003). The patient health questionnaire-2: validity of a two-item depression screener. Med Care.

[35] Arrieta J, Aguerrebere M, Raviola G, Flores H, Elliott P, Espinosa A, Reyes A, Ortiz-Panozo E, Rodriguez-Gutierrez EG, Mukherjee J, Palazuelos D, Franke MF (2017). Validity and utility of the Patient Health Questionnaire (PHQ)-2 and PHQ-9 for screening and diagnosis of depression in rural Chiapas, Mexico: a cross-sectional study. J Clin Psychol.

[36] Bridges AJ, Dueweke AR, Anastasia EA, Rodriguez JH (2019). The positive predictive value of the PHQ-2 as a screener for depression in Spanish-speaking Latinx, English-speaking Latinx, and non-Latinx white primary care patients. J Lat Psychol.

[37] Errazuriz A, Beltrán R, Torres R, Passi-Solar A (2022). The validity and reliability of the PHQ-9 and PHQ-2 on screening for major depression in Spanish speaking immigrants in Chile: a cross-sectional study. Int J Environ Res Public Health.

[38] Sternthal MJ, Slopen N, Williams DR (2011). Racial disparities in health: how much does stress really matter?. Du Bois Rev.

[39] Baciu A, Negussie Y, Geller A, Weinstein JN, National Academies of Sciences Engineering, and Medicine, Health and Medicine Division, Board on Population Health and Public Health Practice, Committee on Community-Based Solutions to Promote Health Equity in the United States (2017). Communities in Action: Pathways to Health Equity.

[40] Araújo BY, Borrell LN (2016). Understanding the link between discrimination, mental health outcomes, and life chances among latinos. Hisp J Behav Sci.

[41] Findling MG, Bleich SN, Casey LS, Blendon RJ, Benson JM, Sayde JM, Miller C (2019). Discrimination in the United States: experiences of latinos. Health Serv Res.

[42] Andrade N, Ford AD, Alvarez C (2021). Discrimination and latino health: a systematic review of risk and resilience. Hisp Health Care Int.

[43] Hooper MW, Nápoles AM, Pérez-Stable EJ (2020). COVID-19 and racial/ethnic disparities. JAMA.

[44] Hwang WC, Goto S (2008). The impact of perceived racial discrimination on the mental health of Asian American and Latino college students. Cultur Divers Ethnic Minor Psychol.

[45] Sanchez D, Chavez FLC, Wagner KM, Cadenas GA, Torres L, Cerezo A (2022). COVID-19 stressors, ethnic discrimination, COVID-19 fears, and mental health among Latinx college students. J Divers High Educ (forthcoming).

[46] Lee YH, Liu Z, Fatori D, Bauermeister JR, Luh RA, Clark CR, Bauermeister S, Brunoni AR, Smoller JW (2022). Association of everyday discrimination with depressive symptoms and suicidal ideation during the COVID-19 pandemic in the all of US Research Program. JAMA Psychiatry.

[47] Held ML, First JM, Huslage M (2022). Effects of COVID-19, discrimination, and social support on Latinx adult mental health. J Immigr Minor Health.

[48] Alegria M, Shrout PE, Woo M, Guarnaccia P, Sribney W, Vila D, Polo A, Cao Z, Mulvaney-Day N, Torres M, Canino G (2007). Understanding differences in past year psychiatric disorders for Latinos living in the US. Soc Sci Med.

[49] Held ML, Lee S (2017). Discrimination and mental health among Latinos: variation by place of origin. J Ment Health.

[50] Jimenez DE, Garza DM, Cárdenas V, Marquine M (2020). Older Latino mental health: a complicated picture. Innov Aging.

[51] García C, Sheehan CM, Flores-Gonzalez N, Ailshire JA (2020). Sleep patterns among US Latinos by nativity and country of origin: results from the national health interview survey. Ethn Dis.

[52] Czeisler MÉ, Lane RI, Petrosky E, Wiley JF, Christensen A, Njai R, Weaver MD, Robbins R, Facer-Childs ER, Barger LK, Czeisler CA, Howard ME, Rajaratnam SMW (2020). Mental health, substance use, and suicidal ideation during the COVID-19 pandemic—United States, June 24-30, 2020. MMWR Morb Mortal Wkly Rep.

[53] Luo Y, Li Q, Jeong H, Cheatham L (2022). The association between social determinants of health and psychological distress during the COVID-19 pandemic: a secondary analysis among four racial/ethnic groups. BMC Public Health.

[54] Baxter T, Shenoy S, Lee HS, Griffith T, Rivas-Baxter A, Park S (2023). Unequal outcomes: the effects of the COVID-19 pandemic on mental health and wellbeing among Hispanic/Latinos with varying degrees of 'Belonging'. Int J Soc Psychiatry.

[55] Noe-Bustamamante L, Gonzalez-Barrera A, Edwards K, Mora L, Hugo LM (2021). Majority of Latinos say skin color impacts opportunity in America and shapes daily life. Pew Research Center.

[56] Molina KM, Alegría M, Mahalingam R (2013). A multiple-group path analysis of the role of everyday discrimination on self-rated physical health among Latina/os in the USA. Ann Behav Med.

[57] Finch BK, Kolody B, Vega WA (2000). Perceived discrimination and depression among Mexican-origin adults in California. J Health Soc Behav.

[58] Sarmiento IA, Cardemil EV (2009). Family functioning and depression in low-income Latino couples. J Marital Fam Ther.

[59] Menselson T, Rehkopf DH, Kubzansky LD (2008). Depression among Latinos in the United States: a meta-analytic review. J Consult Clin Psychol.

[60] Aranda MP, Castaneda I, Lee PJ, Sobel E (2001). Stress, social support, and coping as predictors of depressive symptoms: gender differences among Mexican Americans. Soc Work Res.

[61] Molina KM, Little TV, Rosal MC (2016). Everyday discrimination, family context, and psychological distress among latino adults in the United States. J Community Psychol.

[62] Padilla C, Thomson D (2021). More than one in four Latino and Black households with children are experiencing three or more hardships during COVID-19. Child Trends.

[63] Noe-Bustamamante L, Manuel KJ, Hugo LM (2021). For Latino parents, pandemic has brought challenges in child care and worries about kids’ academic progress. Pew Research Center.

[64] Gould E, Perez D, Wilson V (2020). Latinx workers—particularly women—face devastating job losses in the COVID-19 recession. Economic Policy Institute.

[65] Kneebone E, Holmes N (2016). U.S concentrated poverty in the wake of the great recession. Brookings.

[66] Rewilak J (2017). The impact of financial crises on the poor. J Int Dev.

[67] (2021). United States: pandemic impact on people in poverty. Human Rights Watch.

[68] Solis J, Franco-Paredes C, Henao-Martínez AF, Krsak M, Zimmer SM (2020). Structural vulnerability in the U.S. revealed in three waves of COVID-19. Am J Trop Med Hyg.

[69] Serafini RA, Powell SK, Frere JJ, Saali A, Krystal HL, Kumar V, Yashaswini C, Hernandez J, Moody K, Aronson A, Meah Y, Katz CL (2021). Psychological distress in the face of a pandemic: an observational study characterizing the impact of COVID-19 on immigrant outpatient mental health. Psychiatry Res.

[70] Vilar-Compte M, Gaitán-Rossi P, Félix-Beltrán L, Bustamante AV (2022). Pre-COVID-19 social determinants of health among Mexican migrants in Los Angeles and New York City and their increased vulnerability to unfavorable health outcomes during the COVID-19 pandemic. J Immigr Minor Health.

[71] Koma W, Artiga S, Neuman T, Claxton G, Rae M, Kates J, Michaud J, Kaiser Family Foundation (2020). Low-income and communities of color at higher risk of serious illness if infected with coronavirus.

